# Identification of *MBOAT2* as an Unfavorable Biomarker Correlated with *KRAS* Activation and Reduced CD8^+^ T-Cell Infiltration in Pancreatic Cancer

**DOI:** 10.1155/2022/4269733

**Published:** 2022-05-04

**Authors:** Zhenchong Li, Hongkai Zhuang, Xinming Chen, Yue Zhang, Zuyi Ma, Shujie Wang, Qian Yan, Zixuan Zhou, Shanzhou Huang, Chuanzhao Zhang, Baohua Hou

**Affiliations:** ^1^School of Medicine, South China University of Technology, Guangzhou, 510006 Guangdong Province, China; ^2^Department of General Surgery, Guangdong Provincial People's Hospital, Guangdong Academy of Medical Sciences, Guangzhou 510080, China; ^3^Department of Hepatobiliary Surgery, Sun Yat-sen Memorial Hospital, Sun Yat-sen University, Guangzhou 510080, China; ^4^Guangdong Provincial Key Laboratory of Malignant Tumor Epigenetics and Gene Regulation, Sun Yat-sen Memorial Hospital, Sun Yat-Sen University, Guangzhou 510080, China; ^5^Department of Hepatobiliary Surgery, Shenshan Medical Hospital, Sun Yat-Sen Memorial Hospital, Sun Yat-Sen University, Shanwei 516600, China; ^6^Department of Hematology, The First Affiliated Hospital of Jinan University, Guangzhou 510630, China; ^7^The Second School of Clinical Medicine, Southern Medical University, Guangzhou, 510515 Guangdong Province, China

## Abstract

**Objectives:**

Limited research on the role of membrane-bound O-acyltransferase domain–containing 2 (*MBOAT2*) in cancer biology exists. In particular, the underlying role of MBOAT2 and its potential mechanisms in pancreatic cancer have not yet been explored. Further study of *MBOAT2* could provide new ideas about the carcinogenesis and treatment of pancreatic cancer (PC).

**Methods:**

In the current study, the potential biological and clinical significances of *MBOAT2* were explored by bioinformatics analysis. Real-time quantitative polymerase chain reaction and western blot analysis were performed to determine the level of *MBOAT2* in pancreatic ductal adenocarcinoma (PDAC) cell lines. MTT, colony formation, and Transwell assays and flow cytometry of cell cycle were performed to analyze PDAC cell proliferation, migration, and cycle progression. The potential relationship between *MBOAT2* level and tumor immunity was analyzed using the ESTIMATE algorithm, CIBERSORT algorithm, and single-sample gene set enrichment analysis.

**Results:**

The level of *MBOAT2* was remarkably upregulated in most tumors, especially pancreatic tumors, and was positively correlated with a greater rate of tumor recurrence, higher histologic grade, and worse overall survival. *MBOAT2* overexpression was also closely correlated with the mutation status and expression level of driver genes, especially *KRAS*. Meanwhile, functional enrichment analysis demonstrated that *MBOAT2* might be involved in cell–cell communication; cell cycling; the Ras signaling pathway; and immune-related biological functions such as the leukocyte activation involved in T-cell–receptor signaling pathway, the inflammatory response, and antigen processing and presentation. Furthermore, *in vitro* experiments demonstrated that *MBOAT2* overexpression accelerated PC cell proliferation and migration. *MBOAT2* overexpression also enhanced *CDK2* and *CCNA2* expression, leading to cell cycle progression from the G1 phase to the G2 phase. Lastly, *MBOAT2* overexpression reduced the infiltration level of CD8^+^ T-cells, plasmacytoid dendritic cells, and activated dendritic cells but triggered a high type-2 T helper/type-1 T helper cell ration (Th2/Th1 ration) in PC.

**Conclusion:**

Our findings suggest that *MBOAT2* is a potential protooncogene in PDAC that predicts a poor prognosis and is related to *KRAS* activation and inferior infiltration of CD8^+^ T-cells in PC.

## 1. Introduction

Pancreatic cancer (PC), one of the most malignant tumors, is widely regarded as a cancer highly associated with immunosuppression, resulting in a global cancer-related mortality rate of up to 4.5% in 2015 [1, 2]. The 5-year survival rate for PC is only about 8% [3]. Immunotherapies that enhance or recruit antitumor immune cells into the tumor microenvironment (TME) remain prospective therapeutic strategies for PC [4]. Immune checkpoint inhibitors have been efficaciously used for multiple solid tumors, including melanoma, hepatocellular carcinoma, and non-small-cell lung cancer [5–8]. Nevertheless, these drugs are unable to achieve a satisfactory response in patients with advanced PC because of the shortage of CD8^+^ T-cells in the TME of PC [9–11]. Therefore, it is critical for cancer researchers to gain insight into the molecular mechanisms involved in immunosuppression in PC and to develop more effective immunotherapies to improve the quality of life among patients with PC.

Membrane-bound O-acyltransferase domain–containing 2 (*MBOAT2*), located at chromosome band 2p25.1, was previously found to be associated with the development of hypertrophic obstructive cardiomyopathy, multiple sclerosis, and adrenomyeloneuropathy [12–14]. Limited research on the potential and possible function of *MBOAT2* in cancer biology exists at this time. The study by Badea et al. found that *MBOAT2* was upregulated in PC, suggesting that *MBOAT2* might be associated with PC development [15]. Moreover, Chen et al. reported that *MBOAT2* was one of the DNA methylation-driven genes in the prognostic model for PC, indicating *MBOAT2* might be an unfavorable biomarker [16]. However, the biological role and mechanism of *MBOAT2* in PC, especially whether *MBOAT2* has an impact on tumor immunity, are not clear and need further investigation.

In this study, we first comprehensively analyzed the expression level of *MBOAT2* and its possible correlation with prognosis in different types of cancers, including PC. Subsequently, we assessed the potential biological functions of *MBOAT2* in PC by way of gene set enrichment analysis (GSEA) and pathway enrichment analysis in ConsensuspathDB (http://cpdb.molgen.mpg.de/) [17]. Finally, we explored the potential relationship between the level of *MBOAT2* and immune cell infiltration levels in PC using the ESTIMATE algorithm, CIBERSORT algorithm, and single-sample GSEA (ssGSEA) [18, 19].

## 2. Materials and Methods

### 2.1. Data Acquisition

Messenger RNA (mRNA) level data were recorded according to the number of fragments per kilobase of transcript per million mapped reads, and relevant clinical characteristics of PC were acquired from The Cancer Genome Atlas (TCGA) (https://cancergenome.nih.gov/) database. Information about mutations in *KRAS*, *TP53*, *SMAD4*, and *CDKN2A* in the TCGA PC cohort was obtained from the cBioPortal database (TCGA provisional dataset). Of 177 patients with PC, 171 had an overall survival (OS) time of >1 month. In addition, multiple Gene Expression Omnibus datasets (GSE79668, GSE62452, GSE28735, and GSE60979) were adopted for further research. All of the above datasets were freely obtained using public resources.

### 2.2. *MBOAT2* Expression Analysis

Several datasets were used to explore the expression level of *MBOAT2* in PC. Primarily, the GSE62452 and GSE60979 datasets were used for *MBOAT2* differential expression analysis, but expression data of *MBOAT2* in PC were also acquired from the Oncomine database (https://www.oncomine.org/resource/main.html) and further analyzed. The GSE28735 dataset contains the information of 45 pairs of pancreatic tumors and pancreatic nontumor tissue samples, which were used in this study for paired differential expression analysis. Then, we used the Human Protein Atlas database (http://proteinatlas.org/) to determine the protein level of *MBOAT2*. Moreover, GEPIA datasets (http://gepia.cancerpku.cn/index.html) were used for differential expression analysis of *MBOAT2* in various types of cancer.

### 2.3. Kaplan-Meier Survival Analysis

Survival analysis was conducted to explore the relationship between the *MBOAT2* level and OS of PC patients in the TCGA, GSE62452, and GSE79668 cohorts. Patients in these cohorts were divided into *MBOAT2* low- and high-expression groups, and a Kaplan-Meier survival curve was subsequently produced using the “survminer” package for R (R Foundation for Statistical Computing, Vienna, Austria) [20]. Next, area under the receiver operating characteristic (ROC) curve (AUC) analysis was performed to evaluate the validity and reliability of using the *MBOAT2* level to determine OS in the TCGA, GSE62452, and GSE79668 datasets, with a higher AUC indicating a better predictive effect. The GEPIA database was also used to determine the relationship between the *MBOAT2* level and OS in 33 different types of cancer.

### 2.4. Association between *MBOAT2* Level and Clinicopathological Characteristics in PC

To explore whether *MBOAT2* participates in PC progression, the relationship between *MBOAT2* level and 9 clinicopathological factors (Table 1) was evaluated. *P* < 0.05 was considered to be statistically significant.

### 2.5. Functional Enrichment Analysis

GSEA version 3.0 (http://software.broadinstitude.org/gsea/) was performed to determine differences between the *MBOAT2* high- and low-expression groups in the area of potential biological progression in the TCGA PC cohort [17]. An annotated gene set, c5.all.v6.2.symbols.gmt (Gene Ontology), was obtained from the Molecular Signatures database. *P* < 0.05 and FDR < 25% were considered to be statistically significant. In addition, coexpression genes of *MBOAT2* in the TCGA cohort were selected (Cor > |0.5|, *P* < 0.05) to be input into ConsensuspathDB (release 34) (http://cpdb.molgen.mpg.de/) for pathway enrichment analysis. Here, *P* < 0.01 was considered to be statistically significant [21].

### 2.6. Cell Culture and Transfection

Normal pancreatic epithelial cells (HPDE-6) and pancreatic ductal adenocarcinoma (PDAC) cell lines (BxPC-3, PANC-1, SW1990, and Aspc-1) were purchased from Procell (Wuhan, China). AsPC-1 cells were cultured in Roswell Park Memorial Institute 1640 medium (Gibco Laboratories, Gaithersburg, MD, USA) with 10% fetal bovine serum (FBS) (Gibco Laboratories), and the other cells were cultured in high-glucose Dulbecco's modified Eagle's medium (Gibco Laboratories) with 10% FBS at 37°C and 5% CO_2_.

To construct overexpressed and knocked down cell lines, the empty vector CON335, *MBOAT2* overexpression vector LV-*MBOAT2* (65963-1), the empty vector CON077, MBAOT2-knockdown vector LV-*MBOAT2*-RNAi (87178-1), LV-*MBOAT2*-RNAi (87179-1), and LV-*MBOAT2*-RNAi (87180-1) were purchased from GENE (Shanghai, China) and transfected into PDAC cells. Following puromycin selection, real-time quantitative polymerase chain reaction (RT-qPCR) and western blot (WB) analysis were used to test the overexpression efficiency.

### 2.7. RT-qPCR

The total mRNA of all PDAC cell lines was extracted using the EZ-press RNA purification kit. RT-qPCR was performed 3 times using SYBR Green Pro Taq (AG, Guangzhou, China). The sequences of the primers used are as follows:

- *MBOAT2*: forward 5′-TGAAGGCAGATCATACCATA-3′ and reverse 5′-AAGGACAGCCCACAAACTAA-3′
- ACTB: 5′-GCGTGACATTAAGGAGAAGC-3′ and reverse 5′-CCACGTCACACTTCATGATGG-3′

### 2.8. Western Blot Analysis

Radioimmunoprecipitation assay lysis buffer containing phenylmethylsulfonyl fluoride (P0100; Solarbio) and protease inhibitor (A8260; Solarbio) was used for protein extraction. Protein concentrations were quantified by bicinchoninic acid protein assay (#23235; Thermo Fisher Scientific, Waltham, MA, USA). The same amount of protein (30 *μ*g) was resolved on 10% sodium dodecyl sulfate–polyacrylamide gel electrophoresis gel and then transferred onto a polyvinylidene fluoride (PVDF) membrane. PVDF membranes were subsequently incubated with anti-*MBOT2* (ab121453, 0.2 *μ*g/mL; Abcam, Cambridge, UK), glyceraldehyde-3-phosphate dehydrogenase (ab181602, 1/10000; Abcam), cyclin A1+cyclin A2 (ab185619, 1/1000; Abcam), and *CDK2* (ab32147, 1/2000; Abcam). After 24 hours, the membranes were incubated with the secondary antibody at room temperature for 60 min. Finally, an ultrahigh-sensitivity ECL kit was used for protein visualization.

### 2.9. Cell Proliferation Assays

MTT and colony formation assays were used to detect cell proliferation. For the MTT assays, AsPC-1 and PANC-1 cells were resuspended in 96-well plates at 2 × 10^3^ cells/well and cultured for 0, 24, 48, 72, or 96 hours. When the selected time point was reached, 20 *μ*L of MTT reagent (cat. no. JT343; Genview) was added to each well for further incubation at 37°C under 5% CO_2_ for 2 hours. Then, after 100 *μ*L of dimethylsulfoxide was added to each well and dissolved, the formazan OD value was detected with a microplate reader at the point of absorbance (OD490). For colony formation assays, 2 × 10^3^ cells/well were cultured at 37°C under 5% CO_2_ for 14 days. After the medium was removed, the adherent cells were washed with phosphate-buffered saline 3 times and then dyed with 0.1% crystal violet to observe the colonies (diameter > 0.3 mm).

### 2.10. Transwell Assays

PDAC cells (1.0 × 10^5^) suspended in 200 *μ*L of medium with 10% FBS were plated in a Transwell upper chamber, while 800 *μ*L of medium with 20% FBS was added to the lower chamber. After 24 hours, the upper chamber was cleaned with a cotton swab and washed with phosphate-buffered saline 3 times. Then, 4% paraformaldehyde and 0.1% crystal violet were used for cell fixation and staining, respectively. Images of migrated cells were collected using an inverted microscope.

### 2.11. Flow Cytometry Analysis of the Cell Cycle

PDAC cells were resuspended in 250 *μ*L of phosphate-buffered saline and combined with 750 *μ*L of 100% absolute ethyl alcohol for fixation and then stored for 4 hours at −20°C. Subsequently, the cells were incubated with 500 *μ*L of propidium iodide reagent for 15 min in the dark. The proportion of cells at different stages was then measured by flow cytometry (ModFit version 3.0; Verity Software House, Topsham, ME, USA).

### 2.12. Immune Infiltration Analysis in PC Based on Several Datasets

First, the ESTIMATE algorithm was used to assess the linear relationship between the immune infiltration level and tumor purity in the TCGA PC cohort. PC samples with a high tumor purity showed a lower level of intratumoral immune infiltration [19]. Usually, a higher immune score suggests a greater degree of infiltration. The CIBERSORT algorithm was subsequently adopted to estimate the proportion of 22 immune cell types in different tumor tissues in the TCGA PC cohort [18]. Next, the R package “GSVA” was employed for ssGSEA to evaluate the activity or enrichment levels, functions, or possible pathways of immune cells in PC samples [22]. The following 13 immune-related terms were obtained: CD8^+^ T-cells, regulatory T-cells, cytolytic activity, tumor-associated macrophages, natural killer cells, type-1 T helper (Th1) cell, type-2 T helper (Th2) cells, plasmacytoid dendritic cells (pDCs), dendritic cells (DCs), T-cell costimulation, activated dendritic cells (aDCs), inflammation promoting, and tumor-infiltrating lymphocytes (TILs) [23]. An analysis of the correlation between *MBOAT2* level and immune infiltration in PC was performed with the Pearson correlation coefficients (|Cor| > 0.30, *P* < 0.05). Further, we validated the immune infiltration landscape of PC using ssGSEA in the GSE62452, GSE79668, and GSE60979 cohorts.

### 2.13. Statistical Analyses

All statistical analyses were performed using the R software version 3.5.2 (http://www.r-project.org/) and SPSS version 25.0 (IBM Corporation, Armonk, NY, USA). Graphs were constructed using GraphPad Prism version 9 (GraphPad Software, San Diego, CA, USA). *P* < 0.05 was considered to be statistically significant.

## 3. Results

### 3.1. The Expression of *MBOAT2* in PC and Other Cancers

**Figure 1:**
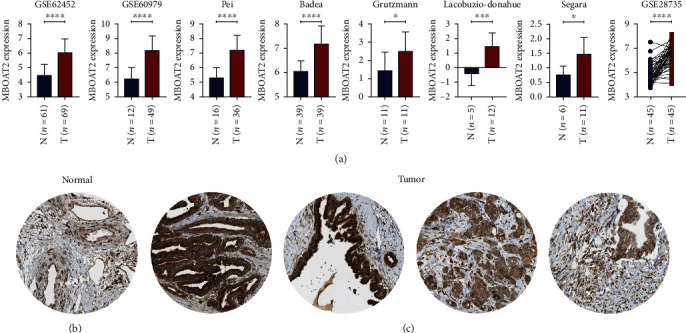
(a) Multiple databases demonstrated that *MBOAT2* is overexpressed in PC. (b) The protein expression of *MBOAT2* in normal pancreatic tissues. (c) The protein expression of *MBOAT2* in PC. *P* values were determined by nonparametric Mann-Whitney *U*-test or two-tailed *t*-tests in (a) (^∗^*P* < 0.05; ^∗∗^*P* < 0.01; ^∗∗∗^*P* < 0.001; ^∗∗∗∗^*P* < 0.0001).

**Figure 2:**
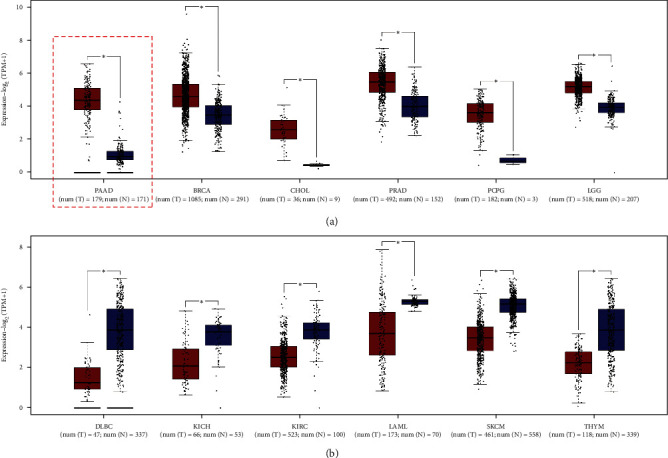
(a) The GEPIA database demonstrated that *MBOAT2* expression is remarkably higher in pancreatic adenocarcinoma (PAAD), breast invasive carcinoma (BRCA), cholangio carcinoma (CHOL), prostate adenocarcinoma (PRAD), pheochromocytoma and paraganglioma (PCPG), and brain lower-grade glioma (LGG). (b) The GEPIA database indicated that the *MBOAT2* level is notably lower in neoplasm diffuse large B-cell lymphoma (DLBC), kidney renal clear cell carcinoma (KIRC), kidney chromophobe (KICH), acute myeloid leukemia (LAML), thymoma (THYM), and skin cutaneous melanoma (SKCM). *P*-values were determined by Non-parametric Mann-Whitney *U*-test in (a) and (b) (^∗^*P* < 0.05).

Primarily, both the GSE62452 and the GSE60979 datasets demonstrated that *MBOAT2* is upregulated in PC tissues compared to human pancreatic nontumor tissues (*P* < 0.0001) (Figure 1(a)). Next, 5 studies of the Oncomine database (e.g., Pei, Badea, Grutzmann, Lacobuzio-Donahue, and Segara) indicated that the level of *MBOAT2* was remarkably higher in PC tissues compared to nontumor tissues (*P* < 0.05) (Figure 1(a)). Besides, a paired differential expression analysis using data from the GSE28735 dataset also proved that the level of *MBOAT2* was relatively higher in PC tissues (*P* < 0.0001) (Figure 1(a)). Furthermore, we used the Human Protein Atlas database to validate the protein expression of *MBOAT2*, and we found that the protein level of *MBOAT2* was significantly upregulated in PC tissues (Figures 1(b) and 1(c)). In addition, using the GEPIA database, we found that the *MBOAT2* level was dramatically higher in pancreatic adenocarcinoma, breast invasive carcinoma, prostate adenocarcinoma, cholangiocarcinoma, pheochromocytoma, paraganglioma, and brain lower-grade glioma (Figure 2(a)). However, *MBOAT2* expression was notably lower in lymphoid neoplasm diffuse large B-cell lymphoma, thymoma, kidney chromophobe, acute myeloid leukemia, kidney renal clear cell carcinoma, and skin cutaneous melanoma (Figure 2(b)). These findings demonstrate that *MBOAT2* is overexpressed in PC and deregulated in other types of cancer.

### 3.2. Prognostic Significance of *MBOAT2* Expression in Human Cancers

**Figure 3:**
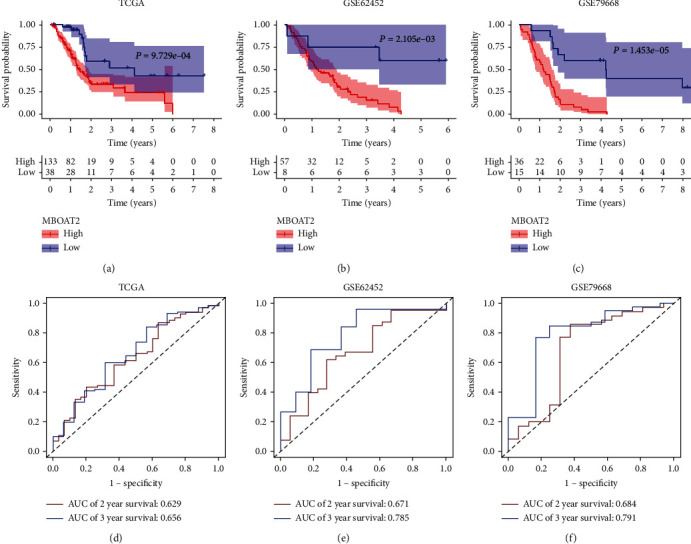
(a–c) *MBOAT2* overexpression was remarkably correlated with worse survival in PC (*P* < 0.05). (d) ROC analysis revealed that the AUCs of *MBOAT2* expression for OS in the TCGA dataset were 0.629 at 2 years and 0.656 at 3 years. (e) ROC analysis revealed that the AUCs of *MBOAT2* expression for OS in the GSE62452 cohort were 0.671 at 2 years and 0.785 at 3 years. (f) ROC analysis revealed that the AUCs of *MBOAT2* expression for OS in the GSE79668 cohort were 0.684 at 2 years and 0.691 at 3 years. *P* values by the log-rank (Mantel-Cox) test are calculated in (a)–(f).

**Figure 4:**
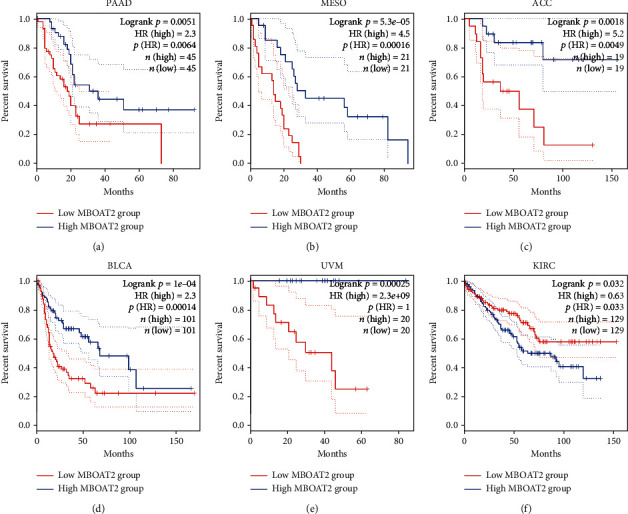
The GEPIA database demonstrated that *MBOAT2* overexpression was closely correlated with poorer prognosis in pancreatic adenocarcinoma, mesothelioma, urothelial carcinoma, adrenocortical carcinoma, and uveal melanoma but was associated with longer survival in kidney renal clear cell carcinoma. *P* values by the log-rank (Mantel-Cox) test are calculated in (a)–(f).

Kaplan-Meier survival analysis using the TCGA, GSE62452, and GSE79668 cohorts revealed that patients with higher levels of *MBOAT2* had a worse OS (*P* < 0.05) (Figures 3(a)–3(c)). The predictive efficacy of *MBOAT2* expression for OS in the TCGA cohort was further verified through ROC analysis, which determined that the AUCs of *MBOAT2* expression for OS in the TCGA dataset were 0.629 at 2 years and 0.656 at 3 years (Figure 3(d)). Meanwhile, in the GSE62452 cohort, the AUCs for OS were 0.671 at 2 years and 0.785 at 3 years (Figure 3(e)), while those in the GSE79668 cohort were 0.684 at 2 years and 0.791 at 3 years (Figure 3(f)). These results indicate that *MBOAT2* has satisfactory performance in the prediction of OS in PC. Furthermore, the GEPIA database demonstrated that *MBOAT2* overexpression was connected to a worse prognosis in pancreatic adenocarcinoma, mesothelioma, uveal melanoma, urothelial carcinoma, and adrenocortical carcinoma but was associated with longer survival in kidney renal clear cell carcinoma (Figure 4). Thus, it can be said that *MBOAT2* overexpression plays a crucial role in the progression of PC. Our findings also demonstrate the prognostic significance of *MBOAT2* expression in several human cancers, though the exact nature of the association may vary by cancer type.

### 3.3. Association between *MBOAT2* Level and the Clinicopathological Characteristics of PC

Details of the association between *MBOAT2* level and the clinicopathological characteristics of PC are shown in Table 1. A high *MBOAT2* level was obviously related to a higher histologic grade (*P* = 0.001) and recurring disease (*P* = 0.006), indicating that *MBOAT2* may play a key role in tumor progression in PC.

### 3.4. Association between *MBOAT2* Expression and Somatic Mutation

**Figure 5:**
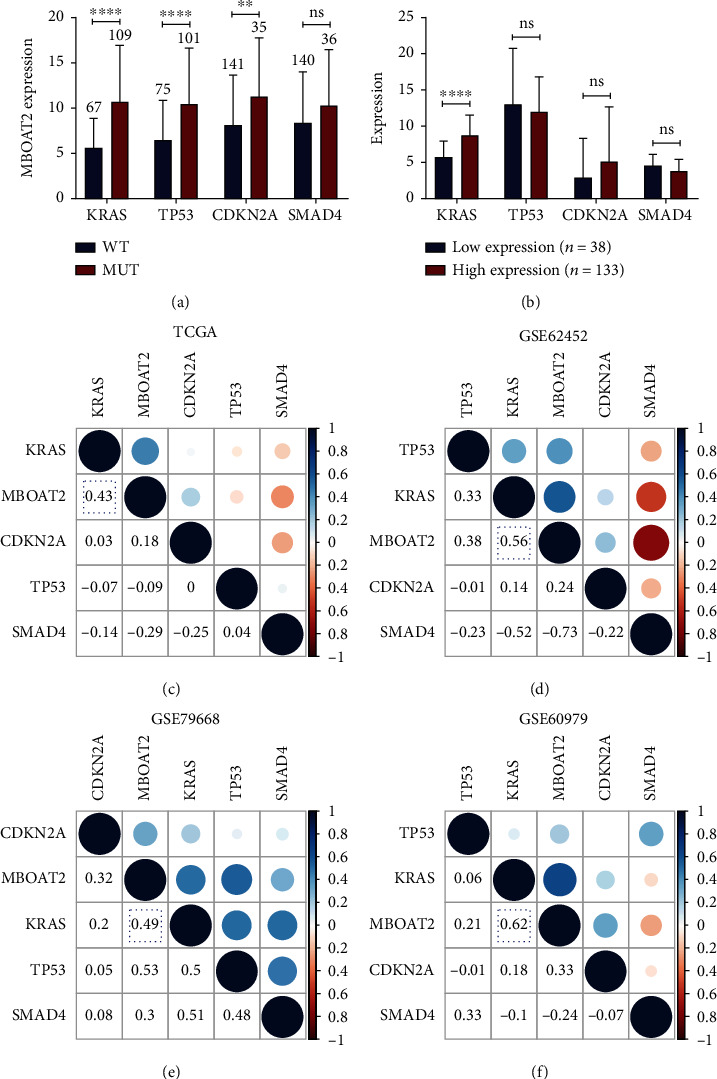
(a) *KRAS*, *TP53*, and *CDKN2A* mutation statuses are dramatically related to a higher expression level of *MBOAT2*. (b) *KRAS* level was notably upregulated in the *MBOAT2* high-expression group (*P* < 0.0001). (c–f) Linear relation analyses indicated that the *MBOAT2* expression level is positively related to that of *KRAS*. *P* values were determined by nonparametric Mann-Whitney *U*-test in (a) and (b) (^∗^*P* < 0.05; ^∗∗^*P* < 0.01; ^∗∗∗^*P* < 0.001; ^∗∗∗∗^*P* < 0.0001).

Our study revealed that *KRAS*, *TP53*, and *CDKN2A* mutation statuses are apparently associated with a higher expression of *MBOAT2*, but the same relationship was not statistically significant for *SMAD4* (Figure 5(a)). We also found that *KRAS* expression was notably upregulated in the *MBOAT2* high-expression group (*P* < 0.0001), but this trend was not statistically significant for *TP53*, *CDKN2A*, or *SMAD4* (Figure 5(b)). In the TCGA cohort, correlation analyses showed that *MBOAT2* level was highly positively correlated with *KRAS* expression (Cor = 0.43, *P* < 0.05), which is very similar as a result to those associated with the GSE62452 cohort (Cor = 0.56, *P* < 0.05), GSE79668 cohort (Cor = 0.49, *P* < 0.05), and GSE60979 cohort (Cor = 0.62, *P* < 0.05) (Figures 5(c)–5(f)). These results indicate that *MBOAT2* overexpression is closely related to the mutation status and the expression of driver genes, especially *KRAS*, thereby suggesting that *KRAS* activation in PC is associated with *MBOAT2* overexpression.

### 3.5. Functional Enrichment Analysis of *MBOAT2* Expression in PC

**Figure 6:**
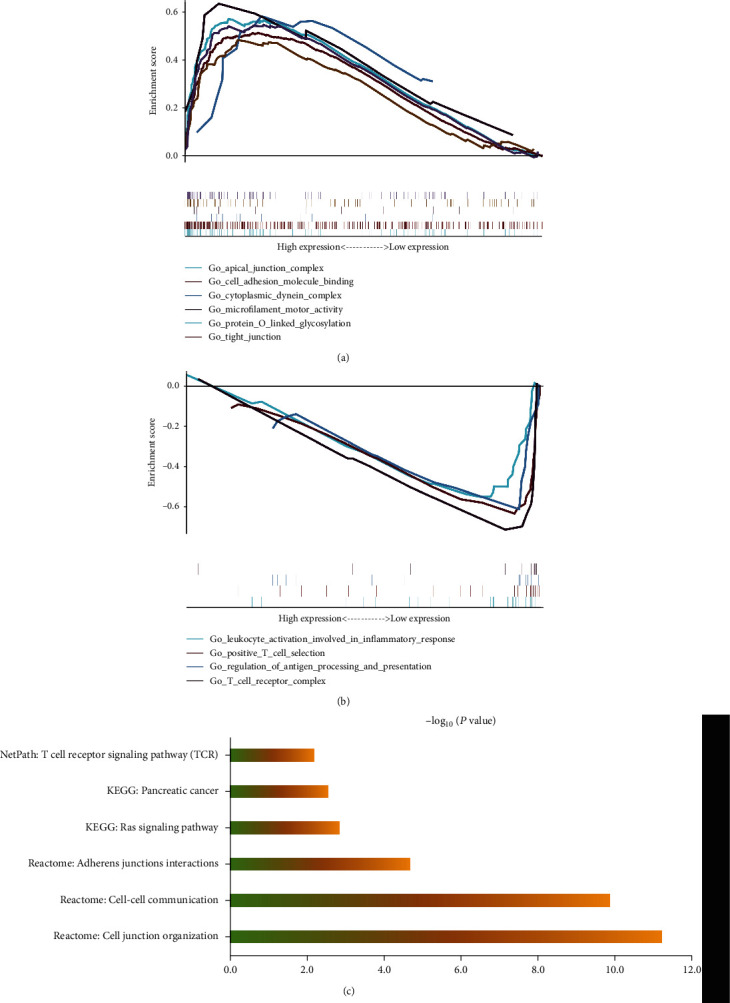
(a) GSEA demonstrated that the apical junction complex–related gene set, cell-adhesion molecular-binding–related gene set, cytoplasmic dynein complex–related gene set, microfilament motor activity–related gene set, protein O-linked glycosylation–related gene set, and tight junction–related gene set were notably enriched in the *MBOAT2* high-expression group. (b) GSEA demonstrated that the leukocyte activation involved in inflammatory response–related gene set, positive T-cell selection, regulation of antigen processing and presentation–related gene set, and TCR complex–related gene set were obviously enriched in the *MBOAT2* low-expression group. (c) Pathway enrichment analysis suggested that *MBOAT2* may participate in PC, the Ras signaling pathway, the TCR signaling pathway, Adherens junction interactions, cell–cell communication, and cell junction organization.

To further explore the role of *MBOAT2* in PC, we analyzed its potential biologically related pathways. GSEA was conducted to discern whether differences in these potential pathways and related genes exist between the *MBOAT2* low- and high-expression groups (Figure 6). We found that the apical junction complex–related gene set, cell-adhesion molecular-binding–related gene set, cytoplasmic dynein complex–related gene set, microfilament motor activity–related gene set, protein O-linked glycosylation–related gene set, and tight junction–related gene set were visibly enriched in the *MBOAT2* high-expression group (Figure 6(a)). Separately, the leukocyte activation involved in inflammatory response–related gene set, regulation of antigen processing and presentation–related gene set, positive T-cell selection, and T-cell receptor (TCR) complex–related gene set were significantly enriched in the *MBOAT2* low-expression group (Figure 6(b)). Using the TCGA PC cohort, we performed a coexpression analysis (Pearson correlation coefficient > 0.5 or < −0.5, *P* < 0.05) for *MBOAT2*. Significantly correlated genes (specifically, 327 coexpression genes in the TCGA PC cohort) were input into ConsensuspathDB (http://cpdb.molgen.mpg.de/) for pathway enrichment analysis (*P* < 0.01) (Figure 6(c)). Subsequently, according to the results of pathway enrichment analysis, *MBOAT2* is probably involved in PC, the Ras signaling pathway, the TCR signaling pathway, Adherens junction interactions, cell–cell communication, and cell junction organization (Figure 6(c)). These results imply that *MBOAT2* overexpression provides essential support for tumor growth and migration and may participate in the regulation of the immune response in PC.

### 3.6. *MBOAT2* Promotes Proliferation and Migration in PDAC

**Figure 7:**
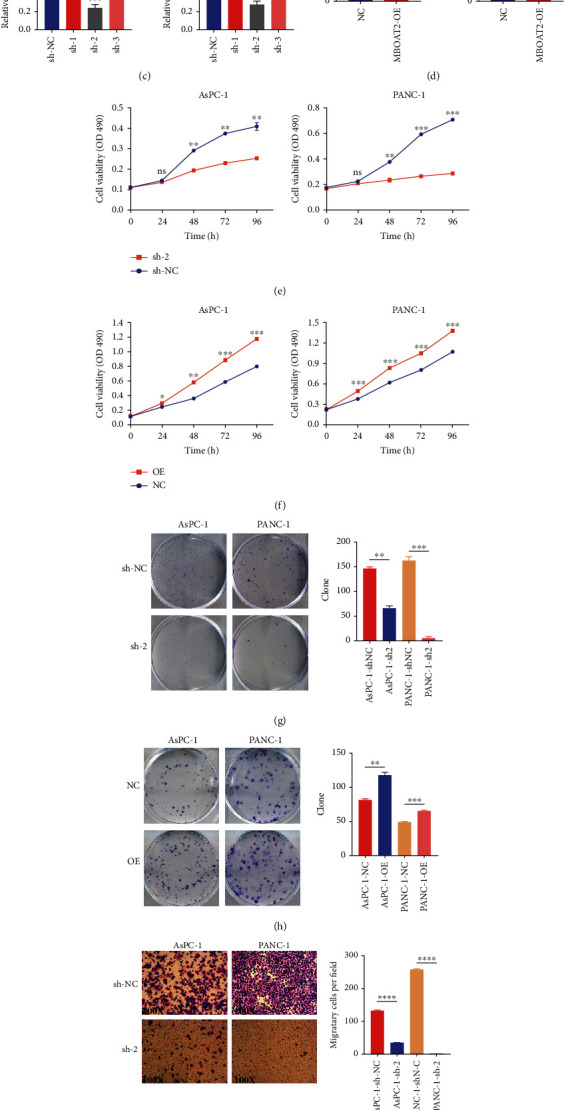
(a and b) RT-qPCR and western blot analysis validated the idea that *MBOAT2* is highly expressed in PDAC cells. (c and d) RT-qPCR confirmed the transfection efficiency of *MBOAT2* in AsPC-1 and PANC-1 cells. (e–h) MTT and colony formation assays showed that *MBOAT2* overexpression promotes PDAC cell proliferation, while *MBOAT2* knockdown represses the proliferation. (i and j) Transwell assay was conducted to evaluate the migration ability of AsPC-1 and PANC-1 cells transfected with LV-*MBOAT2* and LV-*MBOAT2*-RNAi. *P* values were assessed using two-tailed *t*-tests and ANOVA, followed by Dunnett's tests for multiple comparisons in (c)–(j). All data represent the means ± SD from three independent experiments (^∗^*P* < 0.05; ^∗∗^*P* < 0.01; ^∗∗∗^*P* < 0.001; ^∗∗∗∗^*P* < 0.0001).

The mRNA and protein expression levels of *MBOAT2* in several PDAC cell lines and HPDE-6 cells were detected. The expression of *MBOAT2* in PDAC cell lines was obviously higher compared to that in HPDE-6 cells (Figures 7(a) and 7(b)). To further explore the biological function of *MBOAT2* in PDAC, we transfected LV-*MBOAT2*-RNAi and LV-*MBOAT2* into AsPC-1 and PANC-1 cells to obtain stable *MBOAT2*-knockdown and -overexpression cells, respectively. The mRNA levels of *MBOAT2* in these 2 cell groups were then verified by RT-qPCR (Figures 7(c) and 7(d)), and MTT and colony formation assays were additionally carried out on these 2 cell lines to evaluate the cells' proliferation *in vitro*. As expected, the results demonstrated that the downregulation of *MBOAT2* significantly lessened PDAC cell proliferation compared to the empty vector CON077 group (Figures 7(e) and 7(g)), while the results for the overexpression group were opposite those of the empty vector CON335 group (Figures 7(f) and 7(h)). Beyond that, Transwell assays revealed that migratory cells were dramatically decreased in the *MBOAT2*-knockdown group but were augmented remarkably in the *MBOAT2*-overexpression group (Figures 7(i) and 7(j)). These findings signal that *MBOAT2* overexpression significantly promotes cell proliferation and migration in PDAC.

### 3.7. *MBOAT2* Regulates Cell Cycle Progression in PDAC

**Figure 8:**
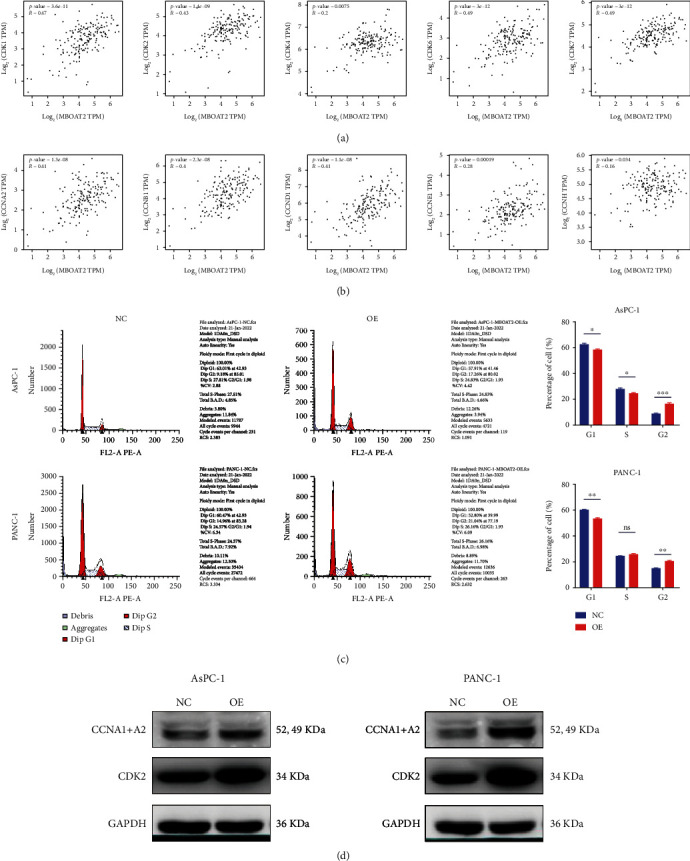
(a and b) Correlations of *MBOAT2* expression levels with the selected 5 CDK and 5 cyclin genes in the GEPIA database. (c) Flow cytometry indicated that overexpression of *MBOAT2* lessened the proportion of G1-phase cells and augmented the percentage of G2-phase cells both among AsPC-1 and PANC-1 cells. (d) Western blot analysis revealed that *MBOAT2* overexpression upregulated the levels of *CDK2* and *CCNA2* in PDAC cells. *P* values were assessed using two-tailed *t*-tests in (c). All figures represent mean ± SD from three independent experiments (^∗^*P* < 0.05; ^∗∗^*P* < 0.01; ^∗∗∗^*P* < 0.001; ^∗∗∗∗^*P* < 0.0001).

Given that cell proliferation may be related to the regulation of the cell cycle, we speculated that *MBOAT2* may be linked to the cell cycle in PDAC. Hence, we used the GEPIA dataset to analyze the linear relationship between *MBOAT2* and 10 cell cycle–associated genes, which predicted that the expression of *MBOAT2* is positively related with *CDK1* (Cor = 0.47, *P* = 3.6e − 11), *CDK2* (Cor = 0.43, *P* = 1.4e − 09), *CDK4* (Cor = 0.2, *P* = 0.0075), *CDK6* (Cor = 0.49, *P* = 3e − 12), *CDK7* (Cor = 0.49, *P* = 3.6e − 12), *CCNA2* (Cor = 0.41, *P* = 1.3e − 08), *CCNB1* (Cor = 0.4, *P* = 2.3e − 08), *CCND1* (Cor = 0.41, *P* = 1.1e − 08), *CCNE1* (Cor = 0.28, *P* = 0.00019), and *CCNH* (Cor = 0.16, *P* = 0.034) (Figures 8(a) and 8(b)). Subsequently, flow cytometry was executed to explore the potential effects of *MBOAT2* on the cell cycle in PDAC, and the results revealed that *MBOAT2* overexpression remarkably reduced the percentage of cells in the G1 phase so that the proportion of G2-phase cells was elevated noticeably both among AsPC-1 and PANC-1 cells, yet there was little change in the amount of S-phase cells in PANC-1 (Figure 8(c)). Due to the linear relationship with *MBOAT*2 > 0.4 and the ability of *CDK2* and *CCNA2* to form the cyclin–CDK complex, we conducted western blot analysis on these 2 genes. Later, we found that the protein expressions of *CDK2* and *CCNA2* were enhanced both in AsPC-1/*MBOAT2*-overexpression cells and PANC-1/*MBOAT2*-overexpression cells compared to the control group (Figure 8(d)). These findings imply that *MBOAT2* can regulate the cell cycle by promoting the formation of the *CCNA2*–*CDK2* complex.

### 3.8. *MBOAT2* Overexpression Suppressed CD8^+^ T-Cell Infiltration in PC

**Figure 9:**
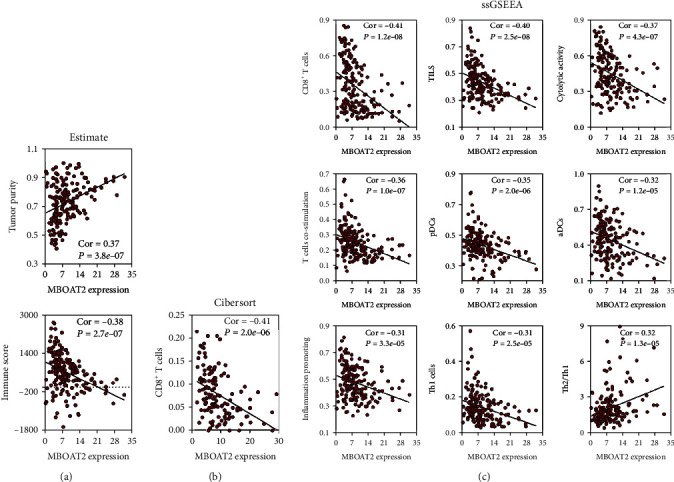
(a) The ESTIMATE algorithm revealed that the *MBOAT2* level was positively correlated with tumor purity (Cor = 0.37, *P* = 3.8e − 07) but negatively correlated with immune score (Cor = −0.38, *P* = 2.7e − 07). (b) The CIBERSORT algorithm revealed that *MBOAT2* overexpression is remarkably connected to the low infiltration level of CD8+ T-cells in the TME of PC (Cor = −0.41, *P* = 2.0e − 06). (c) A correlation analysis between *MBOAT2* level and immune-related terms from the ssGSEA analysis of the TCGA PC cohort (|Cor| > 0.30, *P* < 0.05). Spearman's correlation was performed in (a)–(c).

**Figure 10:**
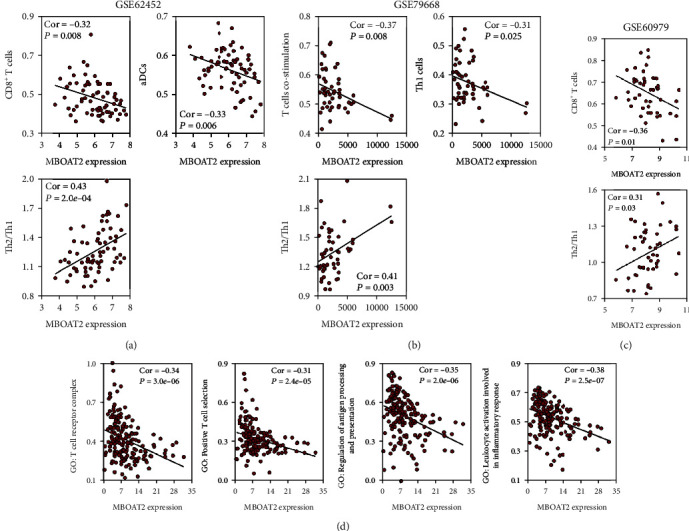
(a) A correlation analysis between the *MBOAT2* level and immune-related terms from the ssGSEA analysis of the GSE62452 cohort (|Cor| > 0.30, *P* < 0.05). (b) A correlation analysis between the *MBOAT2* level and immune-related terms from the ssGSEA analysis of the GSE79668 cohort (|Cor| > 0.30, *P* < 0.05). (c) A correlation analysis between the *MBOAT2* level and immune-related terms from the ssGSEA analysis of the GSE60979 cohort (|Cor| > 0.30, *P* < 0.05). (d) A correlation analysis between the *MBOAT2* level and immune-related biological functions from GSEA of the TCGA PC cohort. Spearman's correlation was performed in (a)–(d).

As shown with the ESTIMATE algorithm, the *MBOAT2* level was positively related to tumor purity (Cor = 0.37, *P* = 3.8e − 07) but negatively correlated with the immune score (Cor = −0.38, *P* = 2.7e − 07) (Figure 9(a)). In addition, the CIBERSORT algorithm reminded us that *MBOAT2* overexpression was noticeably associated with a low level of CD8^+^ T-cell infiltration in the TME of PC (Cor = −0.41, *P* = 2.0e − 06) (Figure 9(b)). The ssGSEA analysis in the TCGA PC cohort indicated that *MBOAT2* expression was negatively associated with the infiltration degree of CD8+ T-cells (Cor = −0.41, *P* = 1.2e − 08), TILs (Cor = −0.40, *P* = 2.5e − 08), cytolytic activity (Cor = −0.37, *P* = 4.3e − 07), T-cell costimulation (Cor = −0.36, *P* = 1.0e − 07), pDCs (Cor = −0.35, *P* = 2.0e − 06), aDCs (Cor = −0.32, *P* = 1.2e − 05), inflammation promoting (Cor = −0.31, *P* = 3.3e − 05), and Th1 cells (Cor = −0.37, *P* = 5.7e − 07) but was positively associated with Th2/Th1 ration (Cor = 0.32, *P* = 1.3e − 05) (Figure 9(c)), similar to the results obtained from the GSE62452 cohort (Cor = −0.32, *P* = 0.008 for CD8^+^ T-cells; Cor = −0.33, *P* = 0.006 for aDCs; and Cor = 0.43, *P* = 2.0e − 04 for Th2/Th1 ration), GSE79668 cohort (Cor = −0.37, *P* = 0.008 for T-cell costimulation; Cor = −0.31, *P* = 0.025 for Th1 cells; and Cor = 0.41, *P* = 0.003 for Th2/Th1 ration), and GSE60979 cohort (Cor = −0.36, *P* = 0.01 for CD8^+^ T-cells and Cor = 0.31, *P* = 0.03 for Th2/Th1 ration) (Figures 10(a)–10(c)). Besides, Gene Set Variation Analysis, a GSVA method that assesses the activities of various pathways in sample populations, was performed to calculate the enrichment scores of the leukocyte activation involved in inflammatory response–related gene set, positive T-cell selection, regulation of antigen presentation and processing–related gene set, and TCR complex–related gene set. Subsequently, the Pearson correlation analysis was carried out to evaluate the linear relationship between *MBOAT2* level and these 4 immune-related functions, which revealed that *MBOAT2* level negatively correlates with the aforementioned 4 kinds of immune-related biological functions (Cor = −0.34, *P* = 3.0e − 06 for the TCR complex; Cor = −0.31, *P* = 2.4e − 05 for positive T-cell selection; Cor = −0.35, *P* = 2.0e − 06 for the regulation of antigen processing and presentation; and Cor = −0.38, *P* = 2.5e − 07 for leukocyte activation involved in inflammatory response, respectively) (Figure 10(d)). These results suggest that *MBOAT2* overexpression impairs the T-cell–related pathway and the antigen presentation pathway, thereby inhibiting the infiltration level and antitumor activity of CD8^+^ T-cells in PC.

## 4. Discussion

Our study demonstrated that the *MBOAT2* level is deregulated in and closely related to the prognosis of several human cancers (Figures 2 and 4). High *MBOAT2* expression correlates with a worse prognosis in several cancers, including PC, adrenocortical carcinoma, mesothelioma, uveal melanoma, and urothelial carcinoma, yet it is associated with longer survival in kidney renal clear cell carcinoma. In addition, the *MBOAT2* level correlates with the pathological degree and recurrence rate in PC patients. Our study suggests that the *MBOAT2* level is an essential and potential prognostic biomarker in various cancers, especially PC.

Through GSEA, we found that biological functions relating to cell–cell communication, such as the apical junction complex–related gene set, cell-adhesion molecular-binding–related gene set, cytoplasmic dynein complex–related gene set, microfilament motor activity–related gene set, protein O-linked glycosylation–related gene set, and tight junction–related gene set, were prominently enriched in the *MBOAT2*-overexpression group (Figure 6(a)). Furthermore, pathway enrichment analysis using the TCGA PC cohort revealed that *MBOAT2* may be involved in PC, the TCR signaling pathway, the Ras signaling pathway, and cell–cell communication (Figure 6(c)). Indeed, *in vitro* experiments revealed that *MBOAT2* overexpression augmented PDAC cell growth and migration, while the results for the *MBOAT2*-knockdown group were opposite (Figures 7(e)–7(j)). In the meanwhile, flow cytometry and WB analysis demonstrated that MBOAT2 overexpression promoted cell cycle progression (Figures 8(c) and 8(d)). Considering these findings, our study has identified *MBOAT2* as a potential protooncogene in PDAC.

Importantly, our study expressed that the *MBOAT2* level negatively correlates with the extent of immune infiltration in PC. Using the ESTIMATE algorithm, we determined that higher levels of *MBOAT2* are associated with greater tumor purity and lower immune scores, indicating that *MBOAT2* overexpression may contribute to tumor progression and immunosuppression (Figure 9(a)). Both the CIBERSORT algorithm and ssGSEA revealed that *MBOAT2* overexpression particularly relates to an inferior infiltration level of CD8^+^ T-cells (Figures 9(b), 9(c), 10(a), and 10(c)), indicating that *MBOAT2* overexpression may lessen the number of CD8^+^ T-cells in the TME of PC. Besides, *MBOAT2* overexpression was closely correlated with lower infiltration of TILs, cytolytic activity, inflammation promoting, and T-cell costimulation. These findings solidify the potential role of *MBOAT2* overexpression in inhibiting TCR signaling, suggesting that *MBOAT2* overexpression suppresses the effects of CD8+ T-cell infiltration and antitumor activity.

Early studies have reported that pDCs and aDCs can present antigens and activate CD8^+^ T-cells through cross-presentation, thereby promoting an immunogenic antitumor response [24–26]. Existing studies also suggest that the tumor antigen presented by DCs is crucial for the induction of antitumor immunity [27–30]. Similarly, we found an obviously positive correlation between cytolytic activity, the infiltration level of DCs, and T-cell costimulation (Figure S1). It has been reported that B-cell receptor signaling played an important role in PC and that B-cells could influence the exclusion of CD8^+^ T-cells through IL35 [31–33]. But there is no obvious correlation between *MBOAT2* level and B-cells from the ssGSEA analysis of the TCGA, GSE62452, GSE79668, and GSE60979 cohort (Figure S2). Our present study demonstrated that *MBOAT2* overexpression may inhibit tumor antigen processing and presentation and correlates with reduced infiltration of aDCs and pDCs. Consequently, we speculated that *MBOAT2* overexpression impairs the antitumor effect of TILs, especially of CD8^+^ T-cells, through suppressing the tumor antigen presentation by DCs (e.g., aDCs and pDCs).

In this study, we discovered that *MBOAT2* overexpression closely correlated with *KRAS* mutation and expression, which further supported the idea that *MBOAT2* is involved in the activation of the Ras signaling pathway. In fact, previous studies have suggested that activation of *KRAS* is beneficial to the establishment of an immunosuppressive environment due to modulating the behavior and even function of immune cells in PC [34]. It was also reported that cancer cells with *KRAS* mutations could recruit myeloid-derived suppressor cells to secrete granulocyte–macrophage colony-stimulating factor, effectively limiting the infiltration level and decreasing the antitumor activities of CD8^+^ cytotoxic T-cells [35]. Other studies have suggested that oncogenic *KRAS* could damage antigen presentation, resulting in the escape of tumor-infiltrating lymphocytes [36]. Taken together, *MBOAT2* overexpression may interact with *KRAS* activation, promoting tumor progression and inhibiting the antitumor effect of CD8^+^ T-cells in PC.

*MBOAT2* overexpression caused high Th2/Th1 ration (Figures 9 and 10). It has been reported that the Th2/Th1 ration is an independent prognostic factor in patients after PC surgery [37–39]. Further, we found that the Th2/Th1 ration negatively correlates with the amount of CD8^+^ T-cells in PC, consistent with the findings of previous studies (Figure S1) [40, 41]. Th1 cells have anticancer properties in the immune system [38, 39]. On the contrary, Th2 cells accelerate the progression of tumor-promoting inflammation, causing patients with PC to have a worse survival outcome [41]. When Th2 is dominant in TME, the infiltration of CD8^+^ T-cells is inhibited or impaired in patients with PC [42]. DeNardo et al. have also put forward the suggestion that Th2 cells could secrete interleukin-4, interleukin-10, and interleukin-13, which weaken the activity of CD8^+^ T-cells [43]. Taken together, our results demonstrate that *MBOAT2* in PC acts as an essential switch in Th1/Th2 ration, which could induce the accumulation of Th2 cells in the TME, resulting in an immunosuppressive TME with an inferior infiltration level of CD8^+^ T-cells. Investigating the molecular mechanism by which *MBOAT2* regulates the immune cell network of Th1, Th2, and CD8^+^ T-cells would be of great significance.

In our study, we focused on the PC immune microenvironment and immunotherapy. We have demonstrated the role of *MBOAT2* in PC and found a novel connection between *MBOAT2* level and immune cell infiltration. However, it is important to note that our study has some flaws. Primarily, this study used publicly available datasets; thus, the study outcomes may be influenced to some extent by the quality of these data. Posteriorly, the potential role of *MBOAT2* in PC and how to modulate immune infiltration in the TME have not been validated *in vivo*. Hence, further *in vivo* experiments and studies should be conducted to illustrate the regulative mechanism between *MBOAT2* and immune cells in PC in the future.

In summary, our findings suggest that *MBOAT2* may be a potential protooncogene in PDAC that predicts poor prognosis and correlates with *KRAS* activation and low infiltration of CD8^+^ T-cells.

## Figures and Tables

**Table 1:** Correlation of MBOAT2 expression to clinicopathological features in PC.

Parameters		MBOAT2 expression		*P*
		Low (*n* = 38)	High (*n* = 133)	
Age	≦60	10 (26.32%)	47 (35.34%)	0.298
	>60	28 (73.68%)	86 (64.66%)	

Gender	Female	20 (52.63%)	58 (43.61%)	0.325
	Male	18 (47.37%)	75 (56.39%)	

AJCC stage	I-IIa	10 (26.32%)	37 (27.82%)	0.884
	IIb-IV	27 (71.05%)	94 (70.68%)	
	Unknown	1 (2.63%)	2 (1.5%)	

Histologic grade	G1	12 (31.58%)	16 (12.03%)	0.001
	G2	16 (42.11%)	76 (57.14%)	
	G3	7 (18.42%)	40 (30.08%)	
	G4	2 (5.26%)	0	
	Unknown	1 (2.63%)	1 (0.75%)	

Recurrence	No	22 (57.89)	44 (33.08%)	0.006
	Yes	16 (42.11%)	89 (66.92%)	

Alcohol history	No	14 (36.84%)	48 (36.09%)	0.769
	Yes	20 (52.63%)	77 (57.89%)	
	Unknown	4 (10.53%)	8 (6.02%)	

Diabetes history	No	18 (47.37%)	87 (65.41%)	0.167
	Yes	10 (26.32%)	26 (19.55%)	
	Unknown	10 (26.32%)	20 (15.04%)	

Tumor size	<4	23 (60.53%)	67 (50.38%)	0.236
	≧4	12 (31.58%)	56 (42.11%)	
	Unknown	3 (7.89%)	10 (7.52%)	

Tumor site	Head	30 (78.95%)	103 (77.44%)	0.073
	Body and tail	2 (5.26%)	25 (18.8%)	
	Unknown	6 (15.79%)	5 (3.76%)	

Note: Statistical significance was calculated by the chi-square test and Fisher's extract test.

## Data Availability

All datasets (TCGA, GSE62452, GSE60979, GSE28735, and GSE79668) are freely available as public resources.

## Supplementary Materials

Supplementary Materials Figure S1: correlation matrix for immune-related terms significantly correlated with *MBOAT2* expression. Figure S2: correlation between *MBOAT2* level and B-cells from the ssGSEA analysis of the TCGA, GSE62452, GSE79668, and GSE60979 cohort. Spearman's correlation was performed in (A-D).
