# Supplementary material for: Identification of MBOAT2 as an Unfavorable Biomarker Correlated with KRAS Activation and Reduced CD8+ T-Cell Infiltration in Pancreatic Cancer
Source: J Oncol. 2022 May 4;2022:4269733. doi: 10.1155/2022/4269733 (PMC9095372; doi:10.1155/2022/4269733)

**Supplementary material**

Figure S1. Correlation matrix for immune-related terms significantly correlated with *MBOAT2* expression.


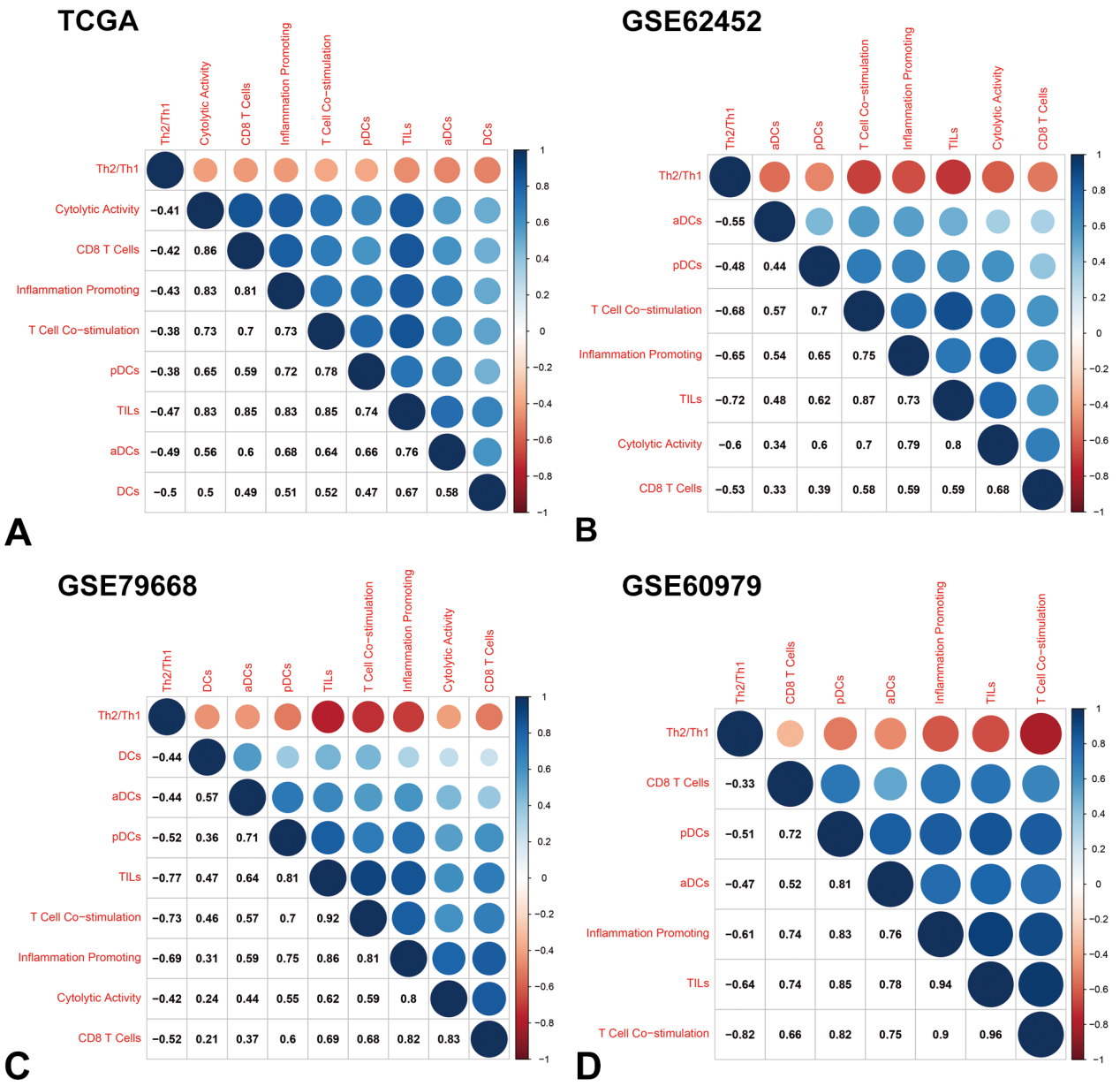


Figure S2. Correlation between *MBOAT2* level and B cells from the ssGSEA analysis of the TCGA, GSE62452, GSE79668 and GSE60979 cohort. Spearman’s correlation was performed in A-D.


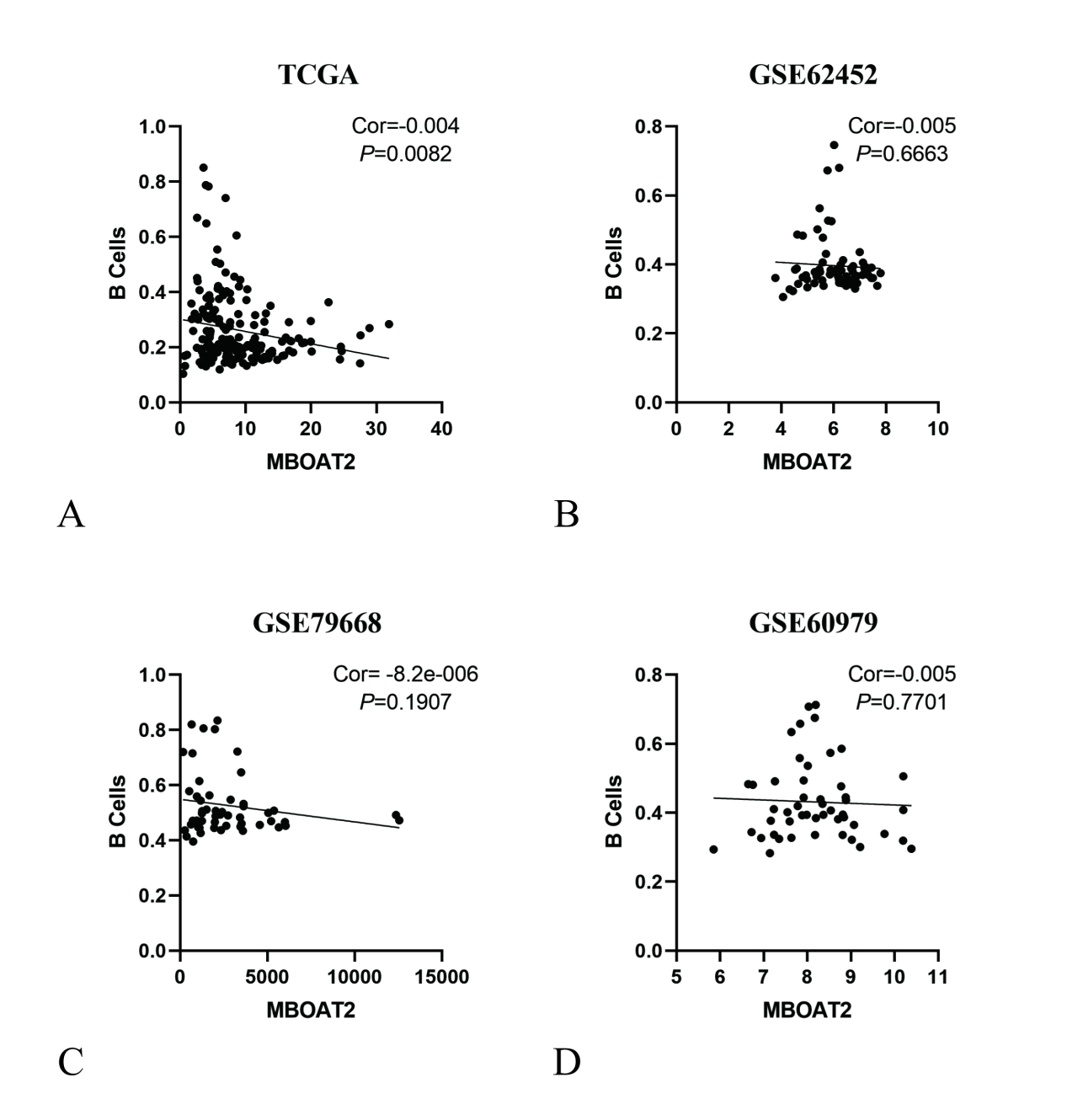

Supplement: Supplementary Materials — Figure S1: correlation matrix for immune-related terms significantly correlated with MBOAT2 expression. Figure S2: correlation between MBOAT2 level and B-cells from the ssGSEA analysis of the TCGA, GSE62452, GSE79668, and GSE60979 cohort. Spearman's correlation was performed in (A-D). [file 4269733.f1.docx]
